# Differential Expression of Granulopoiesis Related Genes in Neutrophil Subsets Distinguished by Membrane Expression of CD177

**DOI:** 10.1371/journal.pone.0099671

**Published:** 2014-06-13

**Authors:** Nan Hu, Helena Mora-Jensen, Kim Theilgaard-Mönch, Berber Doornbos-van der Meer, Minke G. Huitema, Coen A. Stegeman, Peter Heeringa, Cees G. M. Kallenberg, Johanna Westra

**Affiliations:** 1 Department of Medicine, Peking University First Hospital, Beijing, China; 2 The Granulocyte Research Laboratory, Department of Hematology, Rigshospitalet, University of Copenhagen, Copenhagen, Denmark; 3 The Finsen Laboratory, Rigshospitalet & Biotech and Research Innovation Centre, University of Copenhagen, Copenhagen, Denmark; 4 Department of Hematology, Skåne University Hospital, University of Lund, Lund, Sweden; 5 Department of Rheumatology and Clinical Immunology, University Medical Center Groningen, University of Groningen, Groningen, The Netherlands; 6 Department of Nephrology, University Medical Center Groningen, University of Groningen, Groningen, The Netherlands; 7 Department of Pathology and Medical Biology, University Medical Center Groningen, University of Groningen, Groningen, The Netherlands; University of Bern, Switzerland

## Abstract

**Objective:**

Differential gene expression in CD177^+^ and CD177^−^ neutrophils was investigated, in order to detect possible differences in neutrophil function which could be related to the pathogenesis of ANCA-associated Vasculitides (AAV).

**Methods:**

Neutrophils were isolated from healthy controls (HC) with high, negative or bimodal CD177 expression, and sorted into CD177^+^ and CD177^−^ subpopulations. Total RNA was screened for expression of 24,000 probes with Illumina Ref-8 Beadchips. Genes showing differential expression between CD177^+^ and CD177^−^ subsets in microarray analysis were re-assessed using quantitative-PCR. CD177 expression on neutrophil precursors in bone marrow was analyzed using quantitative PCR and flowcytometry.

**Results:**

The proportion of CD177^+^ cells increased during neutrophil maturation in bone marrow. Fold change analysis of gene expression profile of sorted CD177^+^ and CD177^−^ neutrophils resulted in 14 genes with fold change (fc) >3 difference in expression. Interestingly, 10 of these genes have been reported to change significantly in expression during neutrophil maturation, and most of these genes were granule protein (GP) coding genes. mRNA expression levels measured by RT-PCR of a number of these GP, and of PR3 and MPO were higher in the CD177^−^ neutrophil subset in HC, however, particular granule protein amounts were comparable between CD177^+^ and CD177^−^ neutrophil subsets. AAV patients had higher amounts of CD177^+^ neutrophils, but contrary to neutrophils from HC expression of GP-genes was increased, possibly due to activation.

**Conclusion:**

The neutrophil population can be distinguished by membrane expression of CD177 into subsets that are different in expression of GP mRNA but not in GP protein production. GP gene expression is also elevated in AAV patients, which is not explained by skewed distribution of CD177^+^ and CD177^−^ subsets but may be associated with neutrophil activation during on-going inflammation.

## Introduction

Anti-neutrophil cytoplasmic autoantibody (ANCA)-associated vasculitides (AAV) comprise granulomatosis with polyangiitis (GPA), microscopic polyangiitis (MPA) and eosinophilic granulomatosis with polyangiitis (EGPA), which share a spectrum of clinical manifestations reflecting necrotizing damage to small- and medium-sized vessels [1], [2]. The pathogenic role of ANCA in AAV is supported by a large body of *in vitro* and *in vivo* evidence, and the presence of ANCA in the circulation is an important serologic marker for diagnosis and follow-up of AAV [3], [4]. Proteinase 3 (PR3) and myeloperoxidase (MPO), both of which are mainly stored in primary granules of neutrophils, have been identified as ANCA antigens [5]–[7]. Although either specificity can occur with any AAV phenotype, PR3-ANCA are most frequently detected in sera of GPA patients [8].

In resting neutrophils, PR3 is mainly contained in azurophilic granules. However, in many individuals a membrane bound form of PR3 (mPR3) can also be detected in a subset of neutrophils making these accessible for ANCA binding. In the general population, the percentage of mPR3 expressing neutrophils ranges from 0 to 100% and is genetically determined [9], [10]. Within a given individual, the percentage of mPR3^high^ neutrophils is constant in time and is not affected by neutrophil activation, disease activity or therapy [9]–[11].

CD177 is a neutrophil specific, GPI-anchored glycoprotein, compartmentalized in secondary granules [12]. Concurrent with mPR3, CD177 also shows differential expression on the neutrophil surface [13]. It has also been observed that mPR3 co-localizes with CD177 on the neutrophil membrane, and the subpopulation of neutrophils expressing CD177 is identical to that expressing mPR3 [14]. Although the mechanism of mPR3-CD177 interaction has not been clearly demonstrated, CD177 is currently proposed as a receptor of mPR3 on the neutrophil surface [14], [15].

In our previous studies, we have reported that proportions of both mPR3- and CD177-expressing neutrophils are increased in AAV patients and a high percentage of mPR3^high^ neutrophils is a risk factor for relapse in GPA [16]–[18]. These observations indicate that two subsets of neutrophils exist based on CD177 expression and that skewed distribution of these two subpopulations may play a role in the pathogenesis of AAV, although we showed that CD177^+^ and CD177^−^ neutrophils can be equally activated by PR3-ANCA [16]. CD177 has been described to be a counter receptor for platelet endothelial cell adhesion molecule (CD31) on endothelial cells [19], but not for platelets [20]. Recently it was reported that there is no correlation between decreased apoptosis rate of neutrophils in AAV and the proportion of CD177^+^ cells [21], and others showed that low expression of neutrophil CD177 was highly associated with clonal myeloid disorders [22]. Because of the lack of knowledge on the biological function for CD177, we performed a gene microarray-based study to investigate differences between CD177^+^ and CD177^−^ neutrophils, in order to investigate whether there is a pathophysiologic background of an increased CD177^+^/mPR3^high^ neutrophil subset for the pathogenesis of AAV.

## Materials and Methods

### Study populations

For the Illumina microarray study, total RNA from neutrophils was isolated from healthy controls (HC) with negative (0%, CD177^neg^, n = 3), bimodal (40∼70%, CD177^bimodal^, n = 3) and high (>70%, CD177^high^, n = 3) levels of CD177 expression. For the CD177^bimodal^ donors (n = 3), neutrophils were also sorted into CD177^+^ and CD177^−^ subpopulations. For the Illumina microarray study, total RNA was isolated from all of the above-mentioned populations of neutrophils. Genes showing differential expression between CD177^+^ and CD177^−^ subsets in microarray were re-assessed with additionally sorted CD177^+/−^ neutrophils from healthy CD177^bimodal^ donors (n = 7) using quantitative RT-PCR.

Whether imbalanced distribution of CD177^+^ and CD177^−^ neutrophil subsets in AAV leads to altered expression levels of these genes was assessed within the total population of neutrophils from HC (n = 19) and GPA patients (n = 8). Characteristics of patients and HC are listed in Table 1. A diagnosis of GPA was based on the Chapel Hill definitions [1]. ANCA specificity was determined by capture ELISA. Disease severity was quantified using the Birmingham Vasculitis Activity Score (BVAS). Patients included in the current study were in remission (BVAS = 0) and were not receiving treatment when blood samples were drawn for analysis. CRP- and ESR levels were recorded.

**Table 1:** Characteristics of patients and healthy controls included in the analysis of granule proteins-related gene expression by RT-PCR.

	HC	GPA
**Number**	19	8
**Gender (% male)**	58	50
**Age, median (range)**	45 (28–60)	65 (47–79)
**CRP (mg/l), median (range)**	ND	<5 (<5–22)
**ESR (mg/l), median (range)**	ND	16 (2–62)

HC: healthy controls; GPA: Patients with granulomatosis with polyangiitis; CRP: C-reactive protein; ESR: erythrocyte sedimentation rate; ND: not determined.

All patients and controls gave informed written consent and the study was approved by the Medical Ethical Committee of the University Medical Center Groningen. The bone marrow studies were performed at the Department of Hematology, Rigshospitalet, University of Copenhagen (Denmark). All samples were obtained after informed consent had been given, according to guidelines established by the local ethics committee.

### Neutrophil isolation and stimulation

Neutrophils were isolated from heparinized venous blood by centrifugation on Lymphoprep (Axis-Shield, Oslo, Norway) as described previously [16]. To avoid activation, cells were kept on ice and washed with Hanks' balanced salt solution (HBSS) without Ca^2+^/Mg^2+^ (HBSS^−/−^; Gibco/Life technologies, Breda, The Netherlands). Isolated neutrophils were used for cell sorting and subsequently for RNA isolation or western blotting as described below, or were stimulated. For this, the cell suspension was transferred to 6-well-plates and stimulated with 1 µg/ml lipopolysaccharide (LPS; *Escherichia coli* O26:B6, Sigma, St Louis, MO, USA) or 100 ng/ml phorbol-myristate acetate (PMA; Sigma-Aldrich, Zwijndrecht, The Netherlands) at 37°C for 4 hours. Cells incubated with normal medium under the same conditions were included as control.

### Membrane staining and sorting of neutrophils

Isolated neutrophils from healthy volunteers were labeled with a monoclonal antibody against human CD177 (NB1, MEM166; Abcam, Cambridge, UK) following manufacturer's instructions. After washing steps, CD177^+^ and CD177^−^ neutrophils were sorted and collected in ice-cold RPMI by a MoFlo high-speed cell sorter (DakoCytomation). Activation of positively and negatively sorted cells was checked by comparing expression for CD66b (Becton Dickinson, Breda, The Netherlands, nr.561645), CD62L (Biolegend, San Diego, CA, nr. 304822) and CD54 (Becton Dickinson, nr. 555512) by flow cytometry, and no differences were found between the groups (data not shown). For RNA extraction, at least 1×10^7^ cells of each subset were collected.

### RNA isolation and microarray hybridization

Total RNA was isolated from neutrophils using a commercially available kit followed by a DNase digestion step (Qiagen RNeasy mini kit and Qiagen RNase free DNase set, respectively, Qiagen Benelux, The Netherlands). Quality and concentration of RNA samples was assessed with the Experion Automated Electrophoresis System (Bio-Rad Laboratories, Hercules, CA, USA). The RNA samples with quality indicator (RQI) number >7.0 were used for further analysis on expression arrays.

Starting with 200 ng of RNA, the Ambion Illumina TotalPrep Amplification Kit was used for anti-sense RNA synthesis, amplification, and purification, according to the manufacturer's protocol (Applied Biosystems/Ambion, USA). Afterwards, 750 ng of complementary RNA per sample was hybridized to Illumina HumanRef8 Bead-Chips (HumanRef-8_V3_0_R1_11282963_A; Illumina, San Diego, CA, USA) and scanned on the Illumina BeadArray Reader. These microarrays contain 24,000 different probes representing 16,238 different genes; some genes are targeted by more than one probe.

### Microarray data analysis

The initial analysis of processing was performed in the Illumina BeadStudio Gene Expression module v3.2. Quantile normalization and data analysis was done by GeneSpring package version 10.0.0 (Agilent Technologies, Santa Clara, CA, USA). Please refer to our Supplementary list with our raw genetic data and accession numbers for the Genespring database. Only samples were included that passed quality control filtering, which was based on the median probe intensity, the correlation with all other samples and the principal component analysis over the samples. The probes were filtered for further analysis with the criterion that the expression value was present in the upper 75% range of all entities in all of the samples from at least one of the compared groups. Differences in gene expression between the compared groups were considered significant based on a fold change (fc)>2.0 in gene expression.

Functional annotation and pathway enrichment of genes was analyzed using the Kyoto encyclopedia of genes and genome (KEGG) pathways with the GeneCodis functional annotation web based tool [23], [24].

### Quantitative RT-PCR (q-PCR)

RNA was extracted from isolated neutrophils. cDNA was synthesized from 1.0 µg of total RNA. mRNA expression of CD177, MPO, PR3, lipocalin-2, defensin α1, defensin α3, defensin α4, bactericidal/permeability-increasing protein (BPI), cathepsin G and β-actin, was measured in triplicate by the Taqman real-time PCR system (ABI Prism 7900HT Sequence Detection System, Applied Biosystems, Foster City, CA) with specific Taqman primers/probes (Applied Biosystems). Amplification was performed using standard conditions and the amount of target transcript was presented as relative expression (2^−ΔCT^) or fold induction (2^−ΔΔCT^) in comparison to unstimulated controls after being normalized to the expression of β-actin as an endogenous reference.

### CD177 mRNA and protein expression on stages of neutrophil differentiation in bone marrow

Isolation of bone marrow (BM) populations representing successive stages of terminal neutrophil differentiation were performed on bone marrow samples from healthy volunteers from Denmark by Mora-Jensen and co-workers as described previously in detail [25]. These different stages are early promyelocytes (EPM), late promyelocytes (LPM), myelocytes (MY), metamyelocytes (MM), band cells (BC), and PMNs. mRNA expression of CD177 was assessed by real time RT-PCR as described previously [25], β-actin was used to normalize gene-expression. Expression of CD177 on the membrane of different stages was detected with monoclonal anti-CD117 by flow cytometry.

### Measurement of granule proteins by quantitative Western Blot

Cell pellets were suspended with Cell Lysis Buffer (BIOKE, Cell Signaling, The Netherlands) supplemented with 10% of a protease inhibitor cocktail (Sigma-Aldrich). Separation by SDS-PAGE and subsequent western blotting was performed with specific antibodies against CD177 (MEM166; Abcam, Cambridge, UK), PR3 (PR3.G3, house-made), MPO (4A4, Santa Cruz Biotechnology, Germany), cathepsin G (#H00001511-B01, Abnova Gmbh, Germany), BPI (H-130, Santa Cruz Biotechnology, Germany) or defensin α3 (#MA1-35495, Pierce Biotechnology, Rockford, USA) together with anti-beta-actin (#A5060, Sigma-Aldrich) as loading control, followed by detection with IRDye secondary antibodies (800CW and 680LT, Li-COR bioscience, Germany). Membranes were scanned and analyzed using an Odyssey IR scanner using Odyssey imaging software 3.0. The protein of interest was expressed as percentage related to the integrated intensity of the loading control.

### Statistical analysis

In microarray data analysis, a t-test or ANOVA p-value <0.05, which was corrected for multiple testing by the Benjamini-Hochberg method, was considered significant. Results of quantitative RT-PCR and western blotting were presented as means and analyzed for statistical differences using Wilcoxon matched pairs test, Spearman correlation test and Mann-Whitney U-test, performed with GraphPad Prism 4.03 (GraphPad Software, San Diego, CA). Two-tailed p values of <0.05 were considered significant.

### Online Supplemental Material

The supplemental gene list (File S1) describes information of 107 genes with fc>2.0 differences that were significantly different in single gene expression corrected for multiple testing (p<0.05) between CD177^neg^ and CD177^high+bimodal^ populations in the microarray study.

## Results

### Microarray analysis

Gene expression of circulating neutrophils from healthy donors was profiled with Illumina Humanref-8 beadchips. As mentioned before, 5 groups were analyzed separately for two sets of comparison, namely analysis of the total neutrophil population from CD177^neg^, CD177^bimodal^ and CD177^high^ donors and comparison between two sorted neutrophil subsets, CD177^+^ and CD177^−^, from CD177^bimodal^ donors. After initial quality control testing and filtering based on expression levels, one sample from a CD177^neg^ donor was excluded, and 18,448 probes were subjected to further analysis for comparison between the groups with total neutrophil populations differentially expressing CD177 as weel as 15,774 probes screened on sorted neutrophil subsets. Microarray data were further confirmed for low frequency or absence of non-granulocytic cells by undetectable levels of lineage-specific genes highly expressed in T- and B-cells (CD3, CD19), monocytes (M-CSFR), eosinophils (eosinophil peroxidase gene (EPX)) and erythroid cells (glycophorin-A).

When analyzing differentially expressed genes among CD177^neg^, CD177^bimodal^ and CD177^high^ donors of the analyzed transcripts, 472 gene probes showed an fc>2.0 difference in expression level between CD177^neg^ and CD177^high^ donors; 565 showed an fc>2.0 expression difference between CD177^neg^ and CD177^bimodal^ donors; and 284 transcripts displayed an fc>2.0 difference between CD177^high^ and CD177^bimodal^ groups. Among these gene probes, 17 transcripts were significantly different in single gene expression corrected for multiple testing (p<0.05, *Supplementary* *Table 1*, *File S2*). Neutrophils from CD177^high^ and CD177^bimodal^ donors were more similar in gene expression profile compared to CD177^ neg^ donors, Therefore, we combined CD177^high^ and CD177^bimodal^ groups and compared these to CD177^neg^ donors in differential gene expression. We found 107 genes with fc>2.0 differences that were significantly different in single gene expression corrected for multiple testing (p<0.05) between CD177^neg^ and CD177^high+bimodal^ populations. However, no functional pathways or annotation clusters were significantly enriched within these gene entities (*Supplementary gene list, File S1*).

To gain a better overview of gene expression profile of these neutrophil subsets, fc analysis was also performed. This resulted in 14 genes with fc>3.0 differences in expression between the two subsets (*Supplementary* *Table 2*, *File S2*). Interestingly, 10 of these genes have been reported to change significantly in expression during neutrophil maturation, and most of them were granule protein (GP) coding genes. Moreover, all 10 genes, displaying up- or down-regulation in BM-neutrophils compared to early-staged neutrophil precursors, showed accordingly higher or lower expression levels in the CD177^+^ subset as compared to the CD177^−^ subset [26]. We speculated that differential expression of GP-related genes, which also represents different stages in neutrophil maturation, might be one of the features distinguishing the CD177 negative subset from the positive subset. Therefore, expression of 43 GP-related genes was compared between the two subsets. Strikingly, 35 (81%) genes showed higher expression levels in CD177^−^ neutrophils as compared to the CD177^+^ subset. Genes with fc>1.5 differences in expression levels are listed in *Table 2*.

**Table 2:** GP-related genes with fold change>1.5 up-regulated expression in the sorted CD177^−^ neutrophil subset as compared to the CD177^+^ subset.

Gene symbol	FC	Synonym	Definition
**DEFA4**	8.81	HNP-4; HP4; HP-4; DEF4	Defensin, alpha 4, corticostatin.
**DEFA3**	6.74	HNP3; HNP-3; DEF3; HP-3	Defensin, alpha 3, neutrophil-specific.
**DEFA1**	6.35	DEF1; DEFA2; HP-1; HNP-1	Defensin, alpha 1.
**CEACAM8**	4.65	NCA-95; CGM6; CD67; CD66b	Carcinoembryonic antigen-related cell adhesion molecule 8.
**LCN2**	3.24	NGAL	Lipocalin 2.
**CTSG**	2.67	MGC23078; CG	Cathepsin G.
**BPI**	2.30		Bactericidal/permeability-increasing protein.
**TCN1**	2.11	TCI; TC1	Transcobalamin I (vitamin B12 binding protein, R binder family).
**NCF1**	1.77	NOXO2; SH3PXD1A; p47phox; NCF1A	Neutrophil cytosolic factor 1, (chronic granulomatous disease, autosomal 1)
**S100PBP**	1.71	DKFZp313K2325; FLJ12903; S100PBPR	S100P binding protein, transcript variant 2.
**CRISP3**	1.69	MGC126588; SGP28; Aeg2; CRS3; CRISP-3; dJ442L6.3	Cysteine-rich secretory protein 3.
**ELA2**	1.68	HLE; GE; PMN-E; NE; HNE	Elastase 2, neutrophil.
**RPL10A**	1.66	Csa-19; NEDD6	Ribosomal protein L10a.
**LTF**	1.54	HLF2; GIG12	Lactotransferrin.

### Validation of differential expression of granule proteins by q-PCR and Western Blot

**Figure 1:**
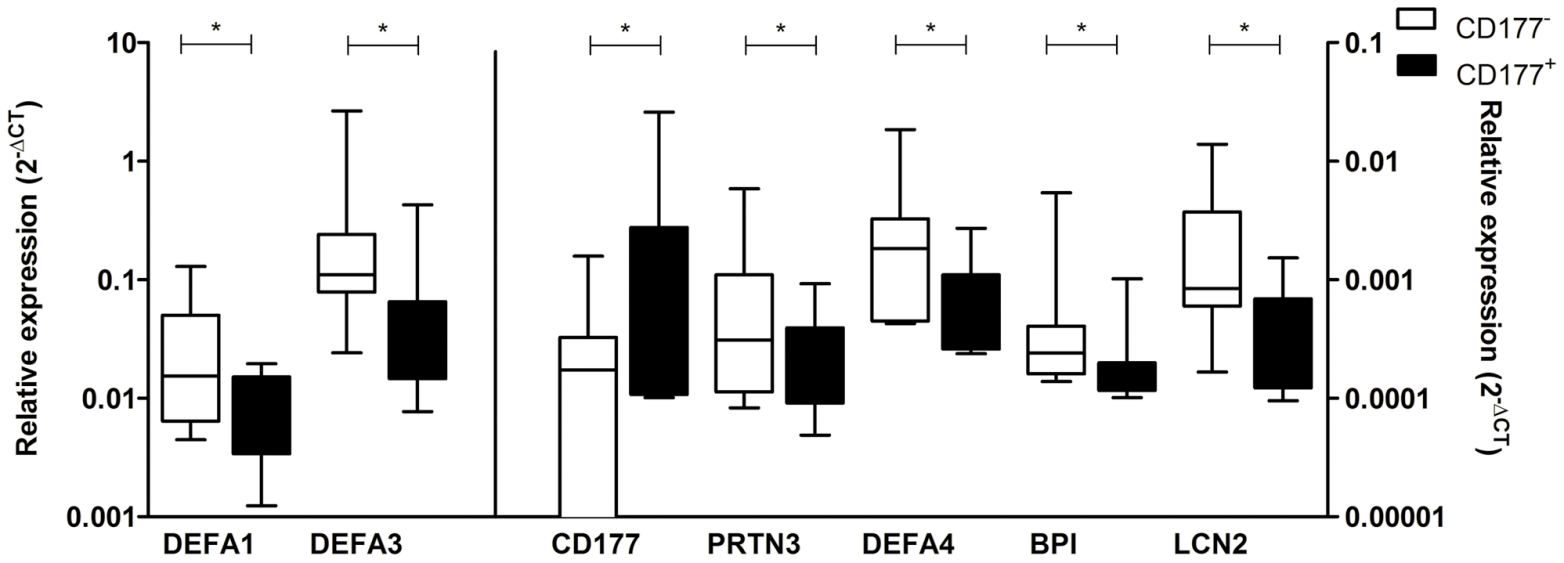
Expression of mRNA levels of graule proteins measured by taqman RT-PCR. Expression levels of mRNA of on left Y-axis defensin α1, defensin α3 (DEFA1, 3), and on right y-axis CD177, PR3 (PRTN3), and defensin α4 (DEFA4), BPI, and lipocalin 2 (LCN2) were measured by q-PCR. Expression levels of GP-related genes in CD177^+^ and CD177^−^ subsets sorted from healthy donors (n = 7) are depicted. *, P<0.05.

To further confirm the findings, expression of some GP-genes was measured as representative examples by q-PCR. Since the sensitivity of microarray chips is considered lower than q-PCR, mRNA levels of PR3, MPO or CD177 were absent from the list of detected probes of microarray analysis, which have, however, been reported to be detected at low levels by q-PCR in healthy persons in other studies [27], [28]. Therefore, as important granule protein genes in AAV, expression of PR3, MPO and CD177 mRNA was also measured by q-PCR. In summary, we analyzed gene expression of 8 granule proteins, most of them stored in azurophilic or specific granules, including CD177, MPO, PR3, defensin α1, α3, and α4, cathepsin G, BPI and lipocalin-2 by q-PCR. Expression of β-actin was tested as endogenous reference. Confirming proper sorting, CD177-mRNA was low/absent from CD177^−^ neutrophils, but expressed at a significantly higher level in the CD177^+^ subset. Results showed that cathepsin-G mRNA was undetectable and MPO expression was low and showed no difference between the two subsets. All the other granule proteins, PR3, defensin α1, α3 and α4, BPI and lipocalin-2, showed a significantly higher mRNA level in CD177^−^ neutrophils than in CD177^+^ cells (*Figure 1*). In the total neutrophil population (n = 19), isolated from healthy donors with different levels of CD177 expression, the percentage of CD177^+^ neutrophils negatively correlated with the mRNA level of BPI (r = −0.6658, p = 0.0027) and defensin α4 (r = −0.4780, p = 0.0384), and showed similar trends of negative correlation with all the other tested GP-genes. In contrast but expected, levels of CD177-mRNA in the total population of neutrophils positively correlated with the proportions of CD177^+^ neutrophils (r = 0.6257, p = 0.0042).

**Figure 2:**
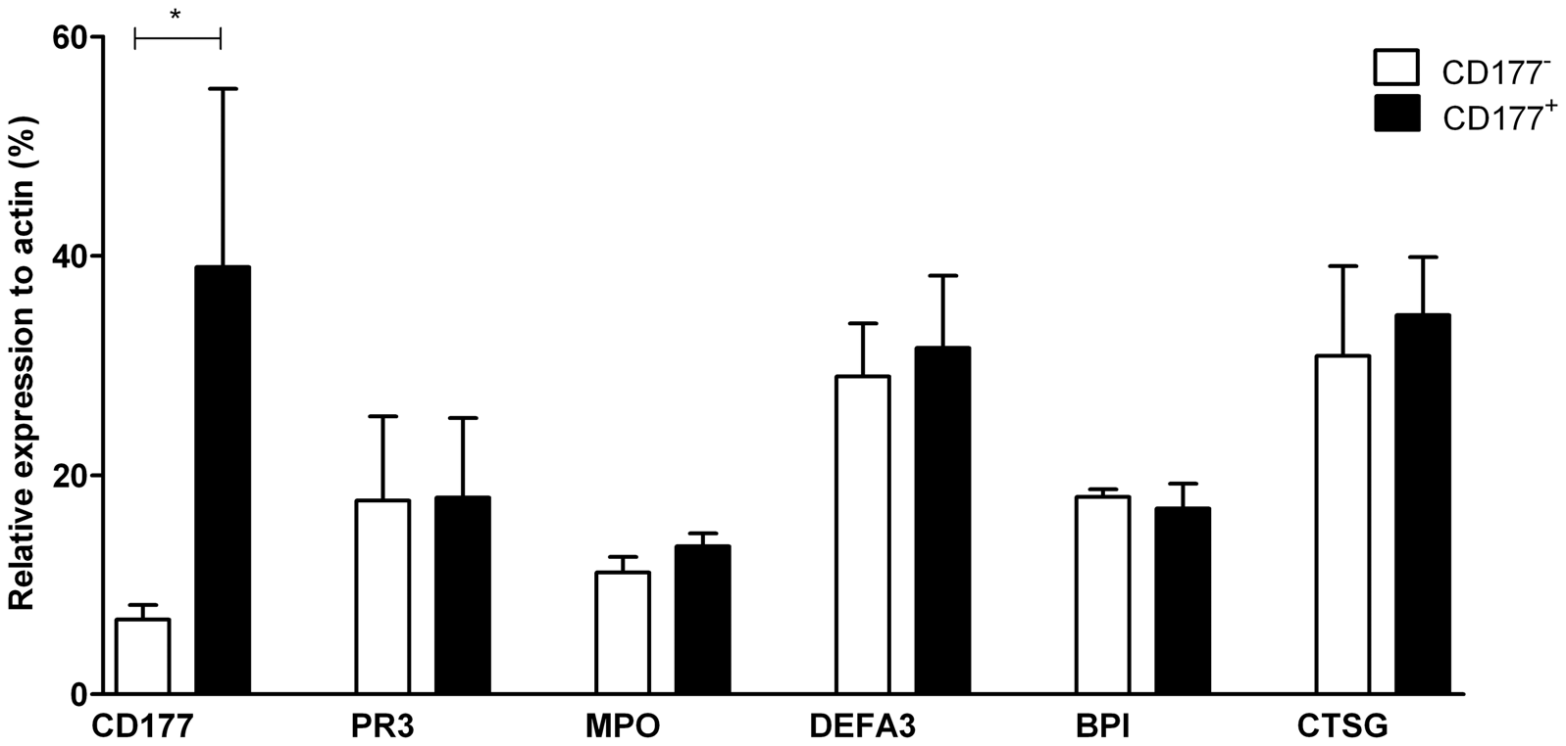
Expression of granule proteins measured by quantitative Western Blot. Expression of CD177, PR3, MPO, defensin α3, BPI and cathepsin G in CD177^+^ and CD177^−^ subsets sorted from healthy donors (n = 5) was measured and quantified by Western Blot (Odyssey). Expression of β-actin was included as loading control for each sample. Results of 5 donors are presented as relative expression, that is percentage as compared to the signal intensity of the loading control.

To assess the differentially expressed GP-related genes at the protein level, we compared the amounts of granule proteins between CD177^+^ and CD177^−^ neutrophil subsets from 5 different donors by quantitative Western Blot. CD177 protein (NB1) was absent in the lysates of membrane-bound CD177 (mCD177) negative neutrophils, but present in the positives. The amounts of GPs, taking PR3, cathepsin G, defensin α3, MPO and BPI as representatives, were comparable between the two neutrophil subsets (*Figure 2*).

### Membrane CD177 expression emerges gradually during neutrophil differentiation

**Figure 3:**
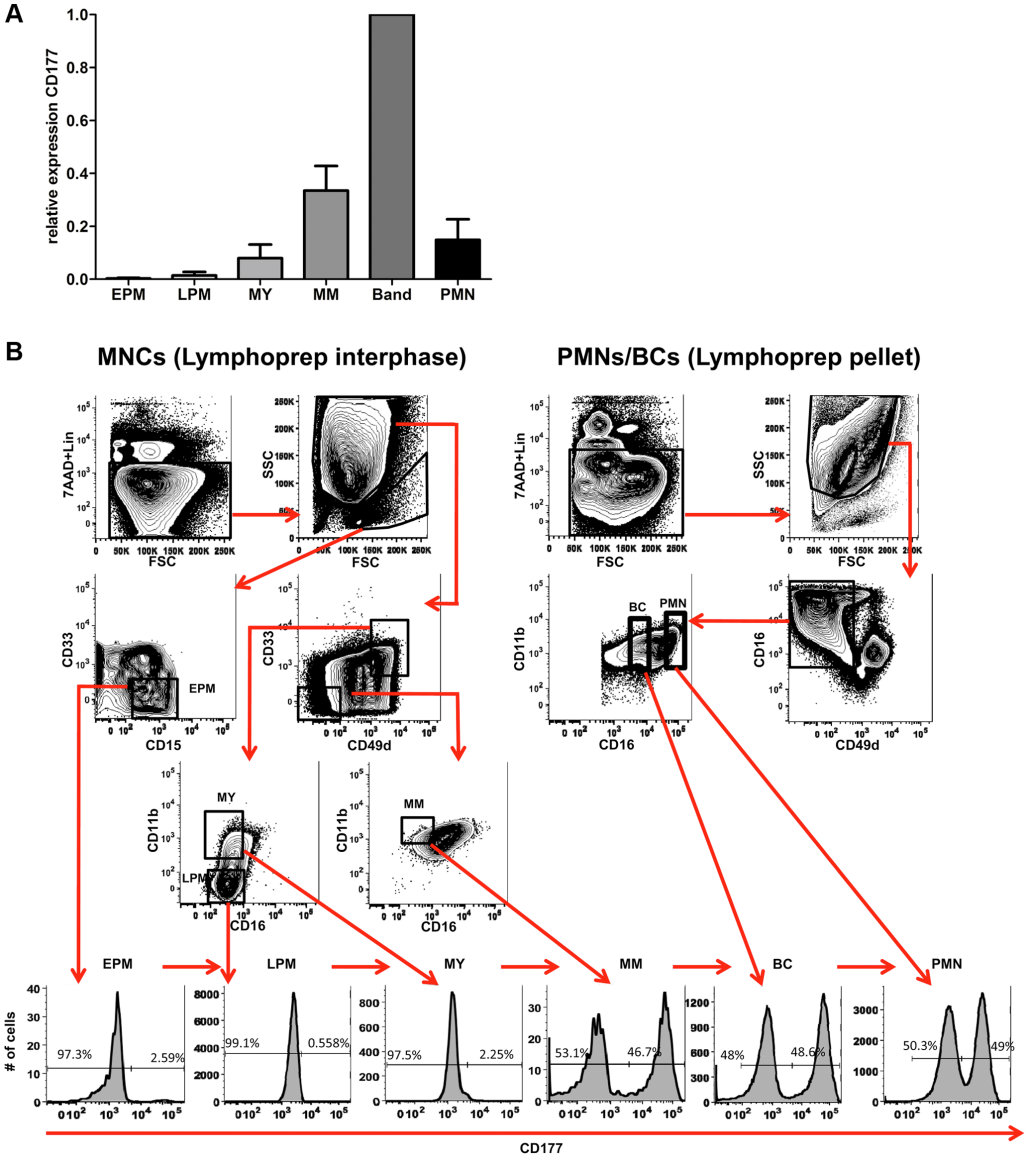
CD177 expression during terminal neutrophil differentiation. BM populations representing successive stages of neutrophil differentiation were isolated from healthy donors by lymphoprep centrifugation followed by immunomagnetic depletion of non-granolocytic cells (MACS) and flow cytometry-based cell sorting. In short, BM was obtained from healthy controls, depleted of erythrocytes and subjected to one-layer density gradient centrifugation using Lymphoprep. The resultant interphase exists of mononuclear cells (MNCs), containing hematopietic stem/progenitor cells and mature cells of all hematopoietic lineages, and the cell pellet contains primarily PMNs and band cells (BCs). Non-granulocytic cells were depleted using a cocktail of biotinylated mAbs followed by incubation with anti-biotin antibody, conjugated with magnetic beads, and run on MACS separation columns (Miltenyi, Bergisch Gladbach, Germany). The resultant lineage-depleted cells are stained with a cocktail of fluorochrome-conjugated MoAbs and streptavidin-PE-Cy5. Addition of streptavidin-PE-Cy5 stains residual non-granulocytic cells that are labeled by biotinylated lineage-specific MoAbs, which allows for additional depletion of non-granulocytic cells during cell sorting. After staining cells were washed and resuspended in PBS/3%FCS containing the DNA dye 7AAD, which allows for sorting of viable non-apoptotic cells (i.e. 7AAD negative cells). Subsequently, the six BM populations representing the successive stages of terminal neutrophil differentiation were sorted using the FACSAria Cell sorter (BD Biosciences, San Jose, CA, USA). (A) mRNA expression of CD177 by quantitative RT-PCR in BM populations from 4 donors was determined and normalized against β-actin. (B) Representative example of CD177 expression on each BM population was measured by flow cytometry. Abbreviations: EPM, early promyelocyte; LPM, late promyelocyte: MY, myelocyte; MM, metamyelocytes; BC, band cells; MNC, mononuclear cells; PMN, polymorphonuclear cells; BM, bone marrow; PB, peripheral blood.

As shown before, GP-related genes, which are supposed to be actively expressed in neutrophil precursors but remarkably down-regulated in mature neutrophils [23], were significantly increased in CD177^−^ neutrophils in a healthy population, suggesting a link between lack of CD177 expression and immature neutrophils. Therefore, CD177 expression was measured during terminal neutrophil differentiation both at the mRNA and the protein level. In figure 3A results of CD177 mRNA levels of 4 different BM donors is shown. Expression of CD177 increased during differentiation and peaked in band cells. CD177 protein expression measured by FACS became evident on stages differentiated to (meta)myelocytes and further. In figure 3B a representative picture is shown from a donor who had 49% CD177 positive mature neutrophils.

### Increased GP gene expression in AAV

**Figure 4:**
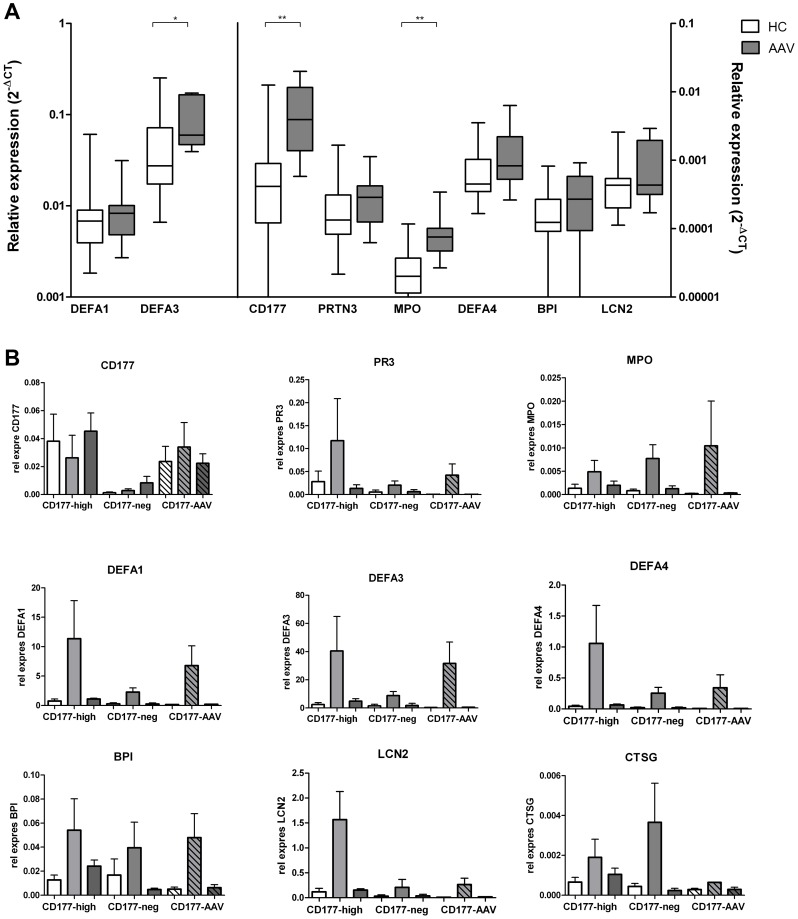
Expression of GP-related genes in AAV. (A) Expression levels of mRNA of defensin α1, α3 (DEFA1,3, left y-axis), and of CD177, PR3 (PRTN3), MPO, defensin α4 (DEFA4), BPI and lipocalin 2 (LCN2) (right y-axis) were measured by q-PCR. Relative expression of GP-mRNA in the total neutrophil population was compared between patients with AAV (n = 8) and healthy donors (n = 19). Horizontal lines denote the median. *, P<0.05; **, P<0.01. (B) Isolated neutrophils of three CD177 negative donors, three CD177 high donors, and three AAV patients were stimulated with PMA or LPS for 4 hours. Total RNA was extracted and quantified for GP gene expression by RT-PCR. Results are presented as relative expression relative to β-actin. White bars are unstimulated samples, grey bars are PMA stimulated samples, dark grey bars are LPS-stimulated samples.

As mentioned before, percentages of CD177^+^ neutrophils are elevated in patients with AAV. This subset shows lower transcription of GP genes than the CD177^−^ subset. It is, therefore, reasonable to assume that circulating neutrophils from AAV patients show a decreased expression of GP genes compared to HC. Total neutrophils were isolated from the peripheral blood of 8 patients with quiescent AAV and 19 healthy donors. Expression of CD177, MPO, PR3, defensin α1, α3, and α4, cathepsin G, BPI and lipocalin-2 was tested and compared between AAV patients and HC. Whereas most of these genes showed comparable or slightly higher levels of GP gene expression compared to HC, CD177-, DEFA3- and MPO- mRNA showed significantly increased expression levels in AAV patients (*Figure 4A*).

Elevated levels of circulating proinflammatory cytokines have been reported in patients with AAV, which may cause in vivo activation of neutrophils [29]. Whether GP gene expression in AAV is influenced by neutrophil activation was subsequently investigated. Neutrophils from three CD177^high^ HC, three CD177^neg^ HC and 3 AAV patients were ex vivo stimulated with PMA or LPS for 4 hours and total RNA was extracted for RT-PCR. Expression of CD177-mRNA was not influenced during activation in all groups compared to unstimulated cells, while transcription of MPO, PR3, lipocalin2, cathepsin G, defensins, and BPI was induced in particular by PMA stimulation. There were no significant differences between the groups at baseline, except for CD177 negative versus CD177 high and between CD177 negative versus AAV (*Figure 4B*).

## Discussion

In this microarray-based study, we investigated differences in gene expression profile between neutrophils from donors with varying levels of mCD177 and between CD177^+^ and CD177^−^ neutrophil subsets from single individuals, in order to investigate the pathophysiologic significance of an increased CD177^+^/mPR3^high^ neutrophil subset for the pathogenesis of AAV.

Based on the data from this microarray analysis neutrophils from donors positive in mCD177 expression, are likely to share a comparable expression profile, but they are different from neutrophils of CD177 negative donors. However, by KEGG pathway analysis, no pathways or annotations of biological functions were significantly enriched when analyzing the genes differentially expressed between donors with and without CD177-expressing neutrophils. Granule protein (GP)-related genes, at the mRNA level, were expressed at higher levels in the CD177^−^ neutrophil subset than in the CD177^+^ subset, the underlying mechanism of which is not clear but may be related to the maturation state of neutrophils. Patients with AAV also displayed increased expression of GP-related genes in their neutrophils, which does not correspond with their higher percentage of CD177^+^ neutrophils compared to HC. On-going inflammation in AAV might explain this observation, since mRNA expression of these GP-related genes could be induced during neutrophil activation.

Mechanisms underlying differential expression of CD177 on neutrophils are not fully understood. Polymorphisms in DNA sequence of CD177, that is, C34G, A778C and G1069A, or methylation in the CpG islands close to the promoter region have been suggested to be associated with a low percentage of CD177^+^ neutrophils in donors with bimodal expression of CD177 [28], [30], [31]. It is known that neutrophil CD177 expression can increase significantly in certain clinical conditions, such as severe bacterial infections and polycythemia vera [32]. Also a significant increased CD177 expression was found in neutrophils from newborns compared to adults, suggesting the existence of additional factors being able to stimulate CD177 expression [33]. Total CD177-deficiency in healthy donors is probably due to an abnormal insertion containing stop codon in the CD177gene [28], [34]. Donors without CD177 expression also displayed different properties compared to CD177-expressing donors in our microarray analysis, which showed 107 gene probes differentially expressed between total neutrophils from CD177-positive donors and donors with CD177 deficiency. However, these genes were described to be associated with various biological functions and no specific biological processes were indicated by the pathway analysis performed with KEGG pathways or GeneCodis functional annotation web-based tool. Besides, no studies have demonstrated abnormal neutrophil function in CD177-deficient healthy donors.

When analyzing the differences between the CD177^+^ and CD177^−^ neutrophil subsets, GP-related genes showed higher expression levels in the CD177^−^ subset than in CD177^+^ neutrophils from the same individual. As a result, GP gene expression negatively correlated with the proportion of CD177-expressing neutrophils in a healthy population. These GP genes are variably expressed during neutrophil differentiation in the bone marrow and highly expressed in neutrophil precursors compared to mature neutrophils [26].

Although circulating immature neutrophils are rarely seen in the normal situation, enriched expression of GP-related genes in both immature neutrophils and the CD177^−^ subset suggests a relationship between CD177^−^ and immature neutrophils. FACS analysis supported this assumption and revealed gradual emergence of CD177 on the membrane of neutrophils during maturation in the bone marrow. CD177 protein expression measured by FACS became evident on stages differentiated to metamyelocytes and further. These data corroborate the data of Stroncek et al, who investigated CD177 expression on fetal bone marrow samples [35] and also those of Meyerson et al, who showed that the most mature neutrophils, expressing CD16 and CD11b also had the highest CD177 expression [22]. Mora-Jensen et al used the same method for isolating neutrophil stages from the bone marrow and investigated the expression of proteins representing the different granules [25]. CD177 expression seems to have the same pattern as MMP-9 which is a tertiary granule protein. When migrated into the blood, these immature neutrophils will be more enriched in the CD177^−^ subset than in the CD177^+^ neutrophil subset, which, to some extent, also explains higher levels of GP gene expression in CD177^−^ neutrophils. Cell functions are mostly dependent on proteins stored or produced within the cell. Although mRNA of GP-related genes was higher expressed in CD177^−^ neutrophils, the amounts of granule proteins, except for CD177, were not significantly different between the two subsets. It has indeed been reported that changes in granule proteins and mRNA expression are not always identical, especially for the early produced proteins during neutrophil maturation, such as proteins stored in azurophilic granules, which are synthesized transiently at the promyelocyte or metamyelocyte stages, and remain stored in granules throughout the terminal granulocytic differentiation [26]. However, not all of the genes showing differential expression between CD177^+^ and CD177^−^ neutrophils were assessed for expression at the protein level. As the major differences between CD177^+^ and CD177^−^ neutrophils were related to GP genes we chose to restrict our analysis at the protein level to granule proteins.

As mentioned, CD177 mRNA expression increased during differentiation of neutrophils, while PR3 and MPO, granule proteins stored in azurophilic granules, had highest mRNA expression in promyelocytes. mRNA expression is absent in mature neutrophils and there is no de novo synthesis of protein in mature neutrophils [26]. So, mRNA expression of CD177 and PR3 is differentially regulated during differentiation of neutrophils. In 2007 von Vietinghoff et al showed that membrane expression of the ANCA antigen PR3 on neutrophils is mediated by CD177 [14]. Abdgawad et al investigated co-expression of mPR3 and CD177 on neutrophils and found that all neutrophils in a given individual were either double-positive or double-negative for PR3 and CD177 [36]. The proportion of double-positive cells was significantly increased in AAV and SLE patients, as our group previously also reported [16]. They further demonstrated increased mRNA levels of both PR3 and CD177 in AAV patients (as shown also in figure 4A), but these levels did not correlate with the proportion of double-positive cells. Furthermore, they showed that when exogenous PR3 was added to CD177 transfected cells, only CD177pos cells bound PR3 to their membrane. Based on these data, the authors concluded that the increased membrane expression of PR3 in AAV is dependent on CD177 expression and correlates with transcription of the CD177 gene [36].

Further considering AAV, patients showing an expanded subset of CD177^+^ neutrophils could supposedly have decreased levels of GP gene expression, as based on the findings in healthy donors. However, our cohort of patients with quiescent AAV showed, increased expression of GP genes, and defensin α4 and MPO expression was significantly higher in these patients compared to controls. This is in line with an earlier study by Yang et al. in which the authors observed significantly increased expression of GP genes in neutrophils from AAV patients, which correlated with disease activity and absolute neutrophil number [37]. It has been suggested that low levels of immune activation exist in AAV patients even in remission [38]. Some of our patients, being in clinical remission, indeed, showed increased levels of CRP and ESR. PMA and LPS induced upregulated expression of these GP genes, suggesting that GP-gene expression is not stable but can be modulated by external stimuli during neutrophil activation. Therefore, on-going inflammation in AAV patients may be responsible for GP gene activation, supported by further increased expression of GP-genes as observed in active AAV patients compared to patients in remission [27], [37]. Whether these overproduced GP-genes are translated into proteins and participate in pathophysiological functions of neutrophils in AAV deserves further investigation.

In summary, the neutrophil population is not homogeneous and can be distinguished by membrane expression of CD177 into subsets which are different in expression of GP-related genes. GP gene expression is also elevated in AAV patients, which is not explained by skewed distribution of CD177^+^ and CD177^−^ subsets, but may be associated with neutrophil activation during on-going inflammation.

## Supporting Information

File S1 **Describes information of 107 genes with fc>2.0 differences that were significantly different in single gene expression corrected for multiple testing (p<0.05) between CD177neg and CD177high+bimodal populations in the microarray study.**
(XLSX)

File S2 **Two supporting tables.**
(DOC)
