# Supplementary material for: Differential Expression of Granulopoiesis Related Genes in Neutrophil Subsets Distinguished by Membrane Expression of CD177
Source: PLoS One. 2014 Jun 13;9(6):e99671. doi: 10.1371/journal.pone.0099671 (PMC4057222; doi:10.1371/journal.pone.0099671)
Supplement: File S2 — Two supporting tables. (DOC) [file pone.0099671.s002.doc]

**Supplementary Table 1. Genes differentially expressed (fc > 2, P < 0.05 corrected for multiple testing by the Benjamini–Hochberg method) among groups with high, bimodal and negative levels of CD177 expression.**

| **Gene symbol** | **Gene Name and Annotation** | **P-value** | **Corrected**  **P-value** | **Fold change** | |
| --- | --- | --- | --- | --- | --- |
|  |  |  |  | H vs. N | B vs. N |
| **ACTRT1** | Actin-related protein T1 . | 8.61E-05 | 0.0145 | -2.36 | -2.14 |
| **DCX** | Doublecortex; lissencephaly, X-linked (doublecortin) (DCX), transcript variant 4. | 1.40E-04 | 0.0145 | -2.13 | -2.11 |
| **EIF1AY** | Eukaryotic translation initiation factor 1A, Y-linked (EIF1AY). | 3.81E-07 | 0.0003 | -26.29 | -1.12 |
| **ENDOGL1** | Endonuclease G-like 1 (ENDOGL1). | 8.59E-04 | 0.0425 | 3.74 | 2.23 |
| **FCGBP** | Fc fragment of IgG binding protein (FCGBP). | 5.01E-06 | 0.0021 | -2.15 | -2.15 |
| **FTSJ1** | FtsJ homolog 1 (E. coli) (FTSJ1), transcript variant 1. | 1.35E-04 | 0.0145 | 2.07 | 3.28 |
| **FXYD5** | FXYD domain containing ion transport regulator 5 (FXYD5), transcript variant 1. | 3.57E-04 | 0.0197 | -2.74 | -2.75 |
| **H2AFJ** | H2A histone family, member J (H2AFJ). | 1.09E-04 | 0.0145 | -2.42 | -3.09 |
| **HIST2H2AA3** | Histone cluster 2, H2aa3 (HIST2H2AA3). | 1.64E-04 | 0.0151 | -2.21 | -2.69 |
| **HIST2H2AC** | Histone cluster 2, H2ac (HIST2H2AC). | 2.42E-04 | 0.0154 | -2.29 | -2.72 |
| **LOC149950** | Hypothetical LOC149950 (LOC149950). | 7.80E-05 | 0.0145 | 2.31 | 1.03 |
| **PYGB** | Phosphorylase, glycogen; brain (PYGB). | 2.29E-04 | 0.0154 | 3.07 | 3.80 |
| **RAMP2** | Receptor (G protein-coupled) activity modifying protein 2 (RAMP2). | 8.71E-04 | 0.0425 | -1.56 | -2.79 |
| **RBM7** | RNA binding motif protein 7 (RBM7). | 2.02E-04 | 0.0152 | 2.14 | 2.53 |
| **RFX3** | Regulatory factor X, 3 (influences HLA class II expression) (RFX3), transcript variant 2. | 1.89E-04 | 0.0152 | -3.38 | -2.82 |
| **RPS4Y1** | Ribosomal protein S4, Y-linked 1 (RPS4Y1). | 1.12E-04 | 0.0145 | -16.59 | -1.03 |
| **SLA** | Src-like-adaptor (SLA), transcript variant 1. | 2.98E-04 | 0.0177 | -3.65 | -4.04 |
| H: high expression level of CD177; B: bimodal expression level of CD177; N: negative expression level of CD177 on circulating neutrophils | | | | | |

**Supplementary Table 2. Genes with fold change>3.0 up/down regulated in the sorted CD177^+^ subset as compared to the sorted CD177^-^ subset of neutrophils from HC.**

| **Gene symbol** | **FC/Regulation** | **Synonym** | **Definition** |
| --- | --- | --- | --- |
| **DEFA4*#** | 8.81/down | HNP-4; HP4; HP-4; DEF4 | Defensin, alpha 4, corticostatin. |
| **LOC728358** | 8.16/down |  | Defensin, alpha 1. |
| **DEFA3#** | 6.74/down | HNP3; HNP-3; DEF3; HP-3 | Defensin, alpha 3, neutrophil-specific. |
| **DEFA1*#** | 6.35/down | DEF1; HP-1; HNP-1 | Defensin, alpha 1. |
| **MS4A3*** | 5.20/down | CD20L; HTM4 | Membrane-spanning 4-domains, subfamily A, member 3. |
| **CEACAM8*#** | 4.65/down | NCA-95; CD67; CD66b | Carcinoembryonic antigen-related cell adhesion molecule 8. |
| **CYBB*#** | 4.12/down | CGD; NOX2; GP91-1 | Cytochrome b-245, beta polypeptide (chronic granulomatous disease). |
| **OLFM4** | 3.98/down | OlfD; GW112; GC1 | Olfactomedin 4. |
| **CEACAM6*#** | 3.95/down | CD66c; NCA; CEAL | Carcinoembryonic antigen-related cell adhesion molecule 6. |
| **RNASE3*#** | 3.73/down | RNS3; ECP | Ribonuclease, RNase A family/3 (eosinophil cationic protein). |
| **JTV1*** | 3.28/down | AIMP2; PRO0992; P38 | JTV1 gene. |
| **LCN2*#** | 3.24/down | NGAL | Lipocalin 2. |
| **ZCCHC11** | 3.22/up | PAPD3 | Zinc finger, CCHC domain containing 11. |
| **CPVL*** | 3.18/down | HVLP; MGC10029 | Carboxypeptidase, vitellogenic-like. |
| N=3. *, Genes showing higher levels of expression in earlier stages of neutrophil maturation. #, genes related to neutrophil granule proteins. | | | |
